# Tangled culprit: a rare case of bezoar-induced small bowel obstruction with pulmonary Edema in a rural Guatemalan woman

**DOI:** 10.1093/omcr/omaf021

**Published:** 2025-04-28

**Authors:** Carlos Diaz, Marcos Orellana, Javier Alarcon, Jose Maaz

**Affiliations:** Department of Research, Universidad Francisco Marroquín, Guatemala City, Guatemala; Department of Research, Universidad Francisco Marroquín, Guatemala City, Guatemala; Department of Research, Universidad Francisco Marroquín, Guatemala City, Guatemala; Department of Research, Universidad Francisco Marroquín, Guatemala City, Guatemala

**Keywords:** Gastroenterology, Respiratory disorders, Critical care medicine, Emergency medicine

## Abstract

We present the unusual case of a 52-year-old woman from a low-resource community in Guatemala who developed small bowel obstruction, followed by acute pulmonary edema. The patient had no prior history of abdominal surgeries or significant comorbidities, making this presentation unexpected. The obstruction was caused by a bezoar and complicated by fluid overload during resuscitation. Radiologic findings revealed dilated bowel loops, suggesting bowel obstruction, and Kerley B lines, indicating concurrent pulmonary edema. This report underscores the challenges of managing complex emergencies in resource-limited settings, highlighting the importance of timely surgical intervention and careful fluid management.

## Introduction

Small bowel obstruction (SBO) is a common abdominal emergency, with adhesions, hernias, and tumors accounting for the majority of cases. However, bezoar-induced obstructions represent a rare etiology, occurring in fewer than 0.4% of all SBO cases [1]. Patients with underlying psychiatric conditions such as pica, as well as those consuming indigestible materials, are more prone to develop bezoars. Although SBO primarily affects the gastrointestinal tract, it can lead to severe systemic complications if left untreated. Pulmonary edema, though uncommon in these cases, may arise secondary to fluid shifts, overaggressive resuscitation, or systemic inflammatory responses.

In patients with SBO, the ‘stack of coins’ sign is a key radiologic finding, reflecting dilated small bowel loops with prominent valvulae conniventes. The presence of acute pulmonary edema in these patients, indicated by Kerley B lines on chest X-ray, is exceedingly rare [2]. This case report details the unusual presentation of a bezoar-induced SBO with pulmonary complications in a woman from a rural area.

## Case presentation

A 52-year-old woman from a rural community in Guatemala presented to the local clinic with severe abdominal pain, distension, and recurrent vomiting over the past 24 h. She reported having eaten a large quantity of dried fruit, after which her symptoms began abruptly. The patient also had a history of pica, with occasional consumption of non-food substances such as paper, but she had never sought medical treatment for this behavior.

Upon arrival, the patient was in respiratory distress, with a respiratory rate of 30 breaths per minute, a heart rate of 120 bpm, and a blood pressure of 80/50 mmHg. Her oxygen saturation was 90% on room air. Abdominal examination revealed marked distension, diffuse tenderness, and hyperactive bowel sounds, raising concern for a mechanical obstruction. A nasogastric tube was inserted, draining copious amounts of bilious fluid.

**Figure 1:**
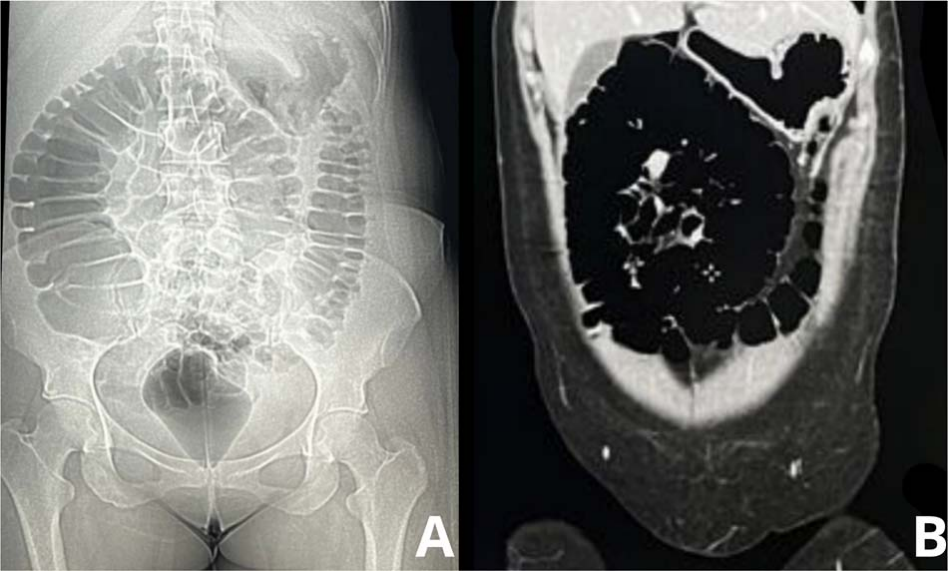
Imaging studies demonstrating ‘Stack of Coins’ sign in small bowel obstruction. A: Plain abdominal radiograph, the abdominal X-ray shows dilated loops of small bowel with visible, closely packed valvulae conniventes, forming the characteristic ‘stack of coins’ appearance. B: Axial CT scan of the abdomen, this contrast-enhanced abdominal CT scan further confirms the small bowel obstruction, with dilated loops filled with gas and fluid.

Abdominal x-rays were performed and a contrast-enhanced abdominal CT scan confirmed dilated loops of small bowel with signs of mechanical obstruction, consistent with an SBO (Fig. 1). A dense intraluminal mass in the jejunum was identified, suggestive of a bezoar, along with free fluid in the peritoneal cavity but no signs of perforation.

**Figure 2:**
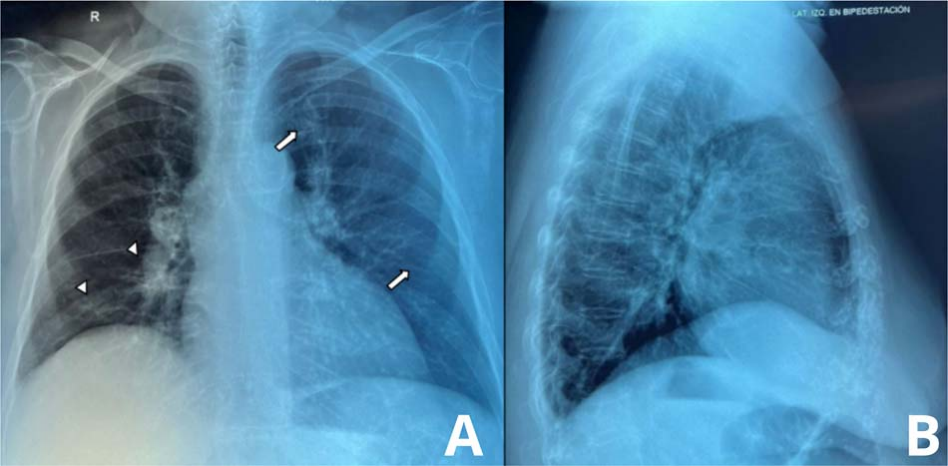
Chest radiograph showing signs of pulmonary Edema. A: Anteroposterior (AP) chest X-ray, this AP chest radiograph demonstrates diffuse interstitial opacities, predominantly in the lower lung fields, consistent with pulmonary edema. Notably, Kerley B lines (white arrows) are visible along the periphery of the lungs. B: Lateral chest X-ray, the lateral view further highlights fluid accumulation along the interlobular septa, contributing to the Kerley B lines.

During resuscitation with intravenous fluids to address her hypotension, the patient’s respiratory status deteriorated. She developed acute dyspnea and pink frothy sputum, and bilateral crackles were heard on lung auscultation. A chest X-ray revealed diffuse alveolar infiltrates and Kerley B lines, indicating acute pulmonary edema (Fig. 2). Despite fluid restriction and supportive measures, her oxygenation worsened, and she required intubation and mechanical ventilation.

The patient underwent urgent exploratory laparotomy with enterotomy, revealing a large bezoar obstructing the jejunum with significant bowel distension. The bezoar was removed, and the bowel was viable without ischemia. Postoperatively, controlled diuresis with intravenous furosemide managed the pulmonary edema, leading to respiratory improvement and extubation within 48 h. She was discharged on postoperative day seven with scheduled psychiatric follow-up for her pica behavior.

## Discussion

This case highlights the rare presentation of bezoar-induced small bowel obstruction (SBO) complicated by acute pulmonary edema. Bezoars are frequently linked to psychiatric conditions like pica, where patients ingest non-food items, or impaired gastric motility. In this patient, significant fluid shifts from the obstruction led to hypovolemia and hypotension. Aggressive fluid resuscitation, intended to treat shock, likely triggered non-cardiogenic pulmonary edema, evidenced by Kerley B lines on chest X-ray. Prompt surgical decompression resolved the SBO, while controlled diuresis supported pulmonary recovery. Given the underlying pica, psychiatric intervention is essential to prevent recurrence and ensure long-term management [3].

On physical examination, percussion of the abdomen typically reveals tympany, while auscultation findings may vary depending on the degree of obstruction. The initial phase of obstruction is characterized frequently by hyperactive bowel sounds, as observed in our patient. In contrast, hypoactive or absent bowel sounds are more indicative of disease progression, often associated with bowel fatigue or perforation. These findings underscore the importance of frequent and careful abdominal examinations in these patients.

The ‘stack of coins’ sign observed on CT imaging reflects dilated small bowel loops with preserved valvulae conniventes, a hallmark of small bowel obstruction [4]. In this case, the obstruction caused significant fluid shifts into the bowel lumen and abdominal cavity, leading to third-spacing of fluids and hypovolemia. Fluid resuscitation is critical in these scenarios, but it must be managed carefully to avoid complications such as pulmonary edema.

Pulmonary edema in this case likely developed due to overzealous fluid administration, exacerbated by the systemic inflammatory response caused by bowel distension. The aggressive fluid resuscitation, intended to correct hypovolemia resulting from third-spacing, overwhelmed the vascular and lymphatic systems, leading to fluid leakage into the alveolar interstitial space. Early initiation of vasopressors in similar cases, particularly those involving septic shock, could be considered to minimize excessive fluid administration and mitigate the risk of pulmonary complications. Although the use of vasopressors in such scenarios remains debated, this case highlights the need to balance fluid resuscitation with hemodynamic support to avoid triggering non-cardiogenic pulmonary edema. The edema, evidenced by Kerley B lines on chest X-ray, resembles acute respiratory distress syndrome (ARDS) but is reversible with appropriate management [5, 6]. Proper treatment includes immediate fluid restriction, controlled diuresis with intravenous furosemide, and respiratory support with oxygen therapy or mechanical ventilation if needed. Additionally, careful hemodynamic monitoring is essential to avoid further complications, ensuring that fluid administration corrects hypovolemia without triggering pulmonary overload [7].

Another important entity worth mentioning is pharmacobezoars, which are conglomerate masses of indigestible tablets that may accumulate within a region of the gastrointestinal tract. This entity should be considered as a differential diagnosis in cases of suspected obstruction, as it can result in intraluminal blockage. The patient’s symptoms often are related with gastric outlet obstruction depending on the site of involvement, due to mechanical accumulation of the bezoar within the lumen and the pharmacologic effects of the substances involved [8]. This dual mechanism highlights an increased risk of drug toxicity that can lead to both surgical and toxicologic emergencies, underscoring the importance of early recognition and management in these cases.

The rural setting posed additional challenges in this case, as limited access to advanced imaging and intensive care delayed definitive treatment. The patient's psychiatric follow-up will also be essential to address her pica behavior and prevent future recurrences.

## Conclusion

This report presents a rare and challenging case of bezoar-induced small bowel obstruction (SBO) complicated by acute pulmonary edema. The bezoar obstructed the gastrointestinal tract, causing fluid shifts that led to third-spacing and hypovolemia. Aggressive resuscitation aimed at stabilizing the patient contributed to fluid overload, triggering non-cardiogenic pulmonary edema, as demonstrated by Kerley B lines on imaging. Early surgical intervention was critical for decompressing the obstructed bowel and preventing further ischemia or necrosis. Postoperative management, including careful diuresis, facilitated respiratory recovery. The patient’s history of pica underscores the importance of psychiatric follow-up to prevent recurrence. This case highlights the need for a multidisciplinary approach in managing complex emergencies.

## Consent

Written consent form was obtained before the inception of this case.

## Data Availability

All data supporting this case report are included in the article, with no additional data available due to patient confidentiality.

## References

[1] Nasri B, Calin M, Shah A. et al. A rare cause of small bowel obstruction due to bezoar in a virgin abdomen. Int J Surg Case Rep 2016;19:144–6. 10.1016/j.ijscr.2015.12.039 26764889 PMC4756184

[2] Gluecker T, Capasso P, Schnyder P. et al. Clinical and radiologic features of pulmonary edema. Radiographics 1999;19:1507–31. 10.1148/radiographics.19.6.g99no211507 10555672

[3] Schnitzler E . The neurology and psychopathology of pica. Curr Neurol Neurosci Rep 2022;22:531–6. 10.1007/s11910-022-01218-2 35674869

[4] Tamburrini S, Serra N, Lugarà M. et al. Ultrasound signs in the diagnosis and staging of small bowel obstruction. Diagn (Basel) 2020;10:277. 10.3390/diagnostics10050277 PMC7277998 32375244

[5] Diamond M, Peniston HL, Sanghavi DK. et al. Acute Respiratory Distress Syndrome. In: StatPearls. StatPearls Publishing: Treasure Island (FL), 2024. 28613773

[6] Loebelenz LI, Ebner L, Obmann VC. et al. Kerley B lines in the lung apex - a distinct CT sign for pulmonary congestion. Swiss Med Wkly 2019;149:w20119. 10.4414/smw.2019.20119 31476240

[7] Lee J, Corl K, Levy MM. Fluid therapy and acute respiratory distress syndrome. Crit Care Clin 2021;37:867–75. 10.1016/j.ccc.2021.05.012 34548138 PMC8449136

[8] Jain SA, Agarwal L, Khyalia A. et al. Pharmacobezoar-a rare case presented as gastric outlet obstruction. J Surg Case Rep 2018;2018:rjy116. 10.1093/jscr/rjy116 29977510 PMC6007504

